# The Major *Fusarium* Species Causing Maize Ear and Kernel Rot and Their Toxigenicity in Chongqing, China

**DOI:** 10.3390/toxins10020090

**Published:** 2018-02-22

**Authors:** Danni Zhou, Xiaoming Wang, Guokang Chen, Suli Sun, Yang Yang, Zhendong Zhu, Canxing Duan

**Affiliations:** 1 Institute of Crop Sciences, Chinese Academy of Agricultural Sciences/National Key Facility for Crop Gene Resources and Genetic Improvement, Beijing 100081, China; zhoudn2014@163.com (D.Z.); wangxiaoming@caas.cn (X.W.); sunsuli@caas.cn (S.S.); yy13269906197@sina.com (Y.Y.); zhuzhendong@caas.cn (Z.Z.); 2 College of Plant Protection, Southwest University, Chongqing 400715, China; chenguokang@swu.edu.cn

**Keywords:** maize, ear and kernel rot, *Fusarium* species, toxigenic genotype, mycotoxin production

## Abstract

*Fusarium verticillioides*, *F. proliferatum*, and *F. meridionale* were identified as the predominant fungi among 116 *Fusarium* isolates causing maize ear and kernel rot, a destructive disease in Chongqing areas, China. The toxigenic capability and genotype were determined by molecular amplification and toxin assay. The results showed that the key toxigenic gene *FUM1* was detected in 47 *F. verticillioides* and 19 *F. proliferatum* isolates. Among these, *F. verticillioides* and *F. proliferatum* isolates mainly produced fumonisin B_1_, ranging from 3.17 to 1566.44, and 97.74 to 11,100.99 µg/g for each gram of dry hyphal weight, with the averages of 263.94 and 3632.88 µg/g, respectively, indicating the *F. proliferatum* isolates on average produced about an order of magnitude more fumonisins than *F. verticillioides* did in these areas, in vitro. Only NIV genotype was detected among 16 *F. meridionale* and three *F. asiaticum* isolates. Among these, 11 *F. meridionale* isolates produced NIV, varying from 17.40 to 2597.34 µg/g. ZEA and DON toxins were detected in 11 and 4 *F. meridionale* isolates, with the toxin production range of 8.35–78.57 and 3.38–33.41 µg/g, respectively. Three *F. asiaticum* isolates produced almost no mycotoxins, except that one isolate produced a small amount of DON. The findings provide us with insight into the risk of the main pathogenic *Fusarium* species and a guide for resistance breeding in these areas.

## 1. Introduction

*Fusarium* species are the main pathogenic fungi causing maize ear and kernel rot worldwide, including *F. verticillioides*, *F. graminearum* species complex (FGSC), *F. oxysporum*, *F. equiseti*, *F. subglutinans* [1,2,3,4]. These pathogens not only cause grain rot, but also produce a variety of mycotoxins that are a direct threat to human and animal health [5,6]. Studies have shown that *F. verticillioides* and *F. proliferatum* mainly produce fumonisin B (FB) that contaminate grains and grain products, whereas members of the FGSC mainly produce trichothecene toxins that contaminate grains. These mycotoxins act as phytotoxins and virulence factors, interact with their hosts [7].

*F. verticillioides* and *F. proliferatum* can produce a variety of secondary metabolites, such as fumonisins and moniliformin in maize-based products [8]. In *Fusarium*-infected maize tissues, FB_1_ predominates, accounting for 75% of the total FB content [9]. Fumonisins have been associated with equine leukoencephalomalacia [10], human esophageal cancer [11], and neural tube defects in newborns [12]. There are several reports on FB contamination in maize in various areas of China. Fu et al. reported that 50% of the maize grains in Hebei, Inner Mongolia, Yunnan, Guizhou, Heilongjiang, Liaoning, and Ningxia provinces were contaminated by FBs [13]. Li et al. analyzed 125 maize samples from Hebei province between 2011 and 2013, of which 46.4% of the samples were contaminated, and the mean contamination levels of FB of the maize samples collected in 2013 reached 706 µg kg^−1^ [14]. FB accumulation in grains is associated with a number of factors, such as toxin production capability of strains [15], host species [16], types of crops [17], and various environmental factors [18].

Currently, 16 phylogenetically distinct species have been identified in the FGSC. The most predominant species associated with small grains diseases are *F. graminearum* sensu stricto, *F. meridionale*, *F. asiaticum*, and *F. boothii* [19,20,21]. The members of FGSC can produce deoxynivalenol (DON), nivalenol (NIV), and other toxins [22]. Based on trichothecene profiles and *Tri13* gene, the FGSC can be divided into three different genotypes: NIV genotype, 3-ADON genotype, and 15-ADON genotype [23]. Previous studies have shown that *F. graminearum* sensu stricto generally belongs to the 15-ADON or 3-ADON genotype [24,25], most of *F. asiaticum* strains belong to the NIV genotype [26] or 3-ADON chemotype [27,28], and the majority of *F. meridionale* strains are of the NIV genotype [29,30,31], but a few belong to 15-ADON or 15-ADON+NIV [32].

The high incidence of maize ear and kernel rot in Chongqing and surrounding areas is mainly due to its special geographical and climatic conditions, as well as cropping systems and resistance level of the major maize cultivars. The incidence of the maize ear and kernel rot is 20–40%, even reaching as high as 75%, which significantly decreases thousand-kernel weight. More seriously, mycotoxin contamination of the affected maize kernels is severe. Up to now, no systematic studies on pathogenic *Fusarium* toxins causing maize ear rot in these areas have been conducted. This study aimed to clarify the composition and distribution of *Fusarium* spp. causing maize kernel rot in Chongqing and surrounding areas, as well as the toxigenic chemotypes and their potentiality and capability. The results will provide effective information on the toxigenic genotype and toxin production capacity of major pathogenic *Fusarium* spp. causing maize kernel rot in the Chongqing and surrounding areas, as well as provide an early warning mechanism for regional maize production.

## 2. Results

### 2.1. Identification of Fusarium spp.

**Figure 1:**
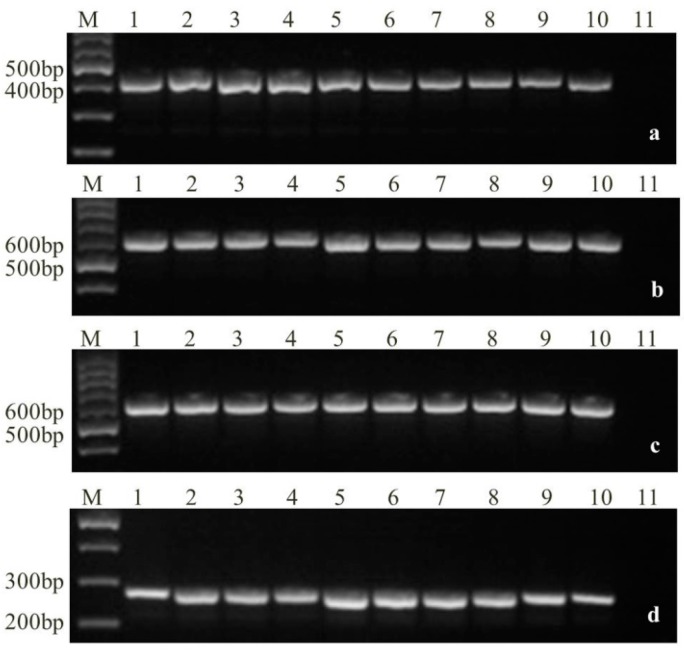
Molecular identification of *Fusarium* species (M, DNA marker; (**a**) PCR amplification of *Fusarium* spp., 1–11: CP1, CP4, CP5, D14, D11, D13, D15, D26, D31, D91, Negative control; (**b**) Specific PCR amplification of *F. verticillioides*, 1–11: D11, D13, D17, D22, D25, D31, D50, D52, D77, D87, Negative control; (**c**) Specific PCR amplification of *F. proliferatum*, 1–11: D21, D57-1, D59, D67, D68-3, D75, D75-2, D79-3, D88-2, D91, Negative control; (**d**) Specific PCR amplification of the FGSC, 1–11: CP1, CP4, CP5, D14, D38, D59-2, D66, D73, D76-1, D99, Negative control).

Based on morphological and molecular findings, a total of 116 *Fusarium* isolates and 10 *Fusarium* species were obtained and identified, including *F. verticillioides*, *F. proliferatum*, FGSC, *F. oxysporum*, *F. fujikuroi*, *F. equiseti*, *F. culmorum*, *F. incarnatum*, *F. kyushuense*, and *F. solani*, with the isolation frequencies of 40.2% (47), 16.4% (19), 16.4% (19), 12.1% (14), 6.9% (8), 3.4% (4), 1.7% (2), 0.9% (1), 0.9% (1), and 0.9% (1), respectively (Table 1 and Figure 1).

There were a few differences in the frequency of *Fusarium* isolates in different regions of Chongqing (Table 2). In Southeast Chongqing, the frequency of *F. verticillioides*, *F. proliferatum*, FGSC, and *F. oxysporum* was 42.86%, 10.07%, 14.29%, and 25.00%, respectively. Therefore, *F. verticillioides* and *F. oxysporum* were the predominant *Fusarium* species in Southeast Chongqing. However, *F. oxysporum* were not be found in West Chongqing. The conclusion should not be drawn for the Central Chongqing and the other regions, due to the smaller sample size.

**Figure 2:**
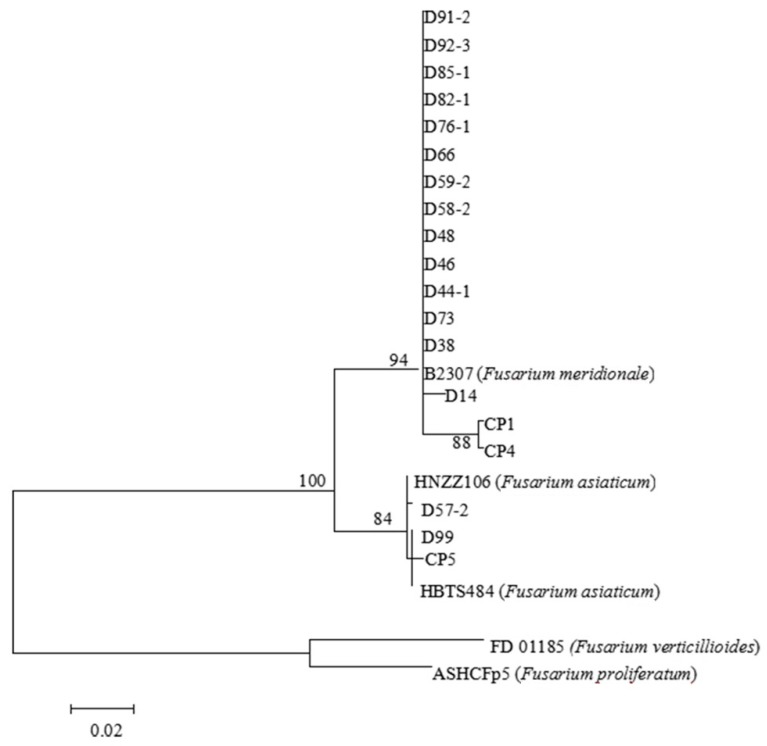
The phylogenetic tree of *F. graminearum* species complex (FGSC) isolates based on *TEF-1α* gene sequences.

Analysis of the sequences of the *TEF-1α* gene of 19 FGSC isolates and alignment with BLAST in the *Fusarium* Center’s database indicated that 19 FGSC isolates contained 16 *F. meridionale* and 3 *F. asiaticum*, with the total isolation frequencies of 15.5% and 2.9%. A total of 16 isolates, such as D38, D46 and others, exhibited 99% to 100% homology with reference strain B2307 (*F. meridionale*), while CP5, D57-2 and D99 showed 99% to 100% homology with reference strains HNZZ106 and HBTS484 (*F. asiaticum*). The tree topologies of the *TEF-1α* gene sequences showed that the classification divided FGSC into two distinct clades, with high clade support values (Figure 2).

### 2.2. Detection of Toxigenic Genes and Chemotypes

**Figure 3:**
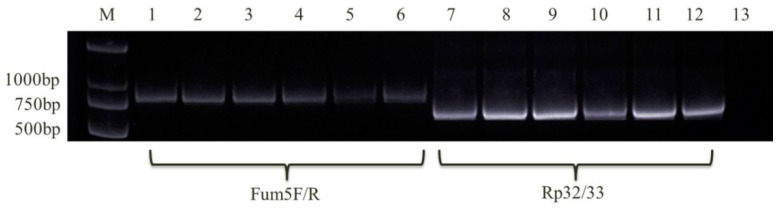
Detection of the key toxigenic gene *FUM1* in *F. verticillioides* isolates using primers Fum5F/Fum5R and Rp32/Rp33 (M, DNA marker; 1–6: D11, D12-1, D12-2, D13, D15, D17; 7–13: D11, D12-1, D12-2, D13, D15, D17, Negative control).

**Figure 4:**
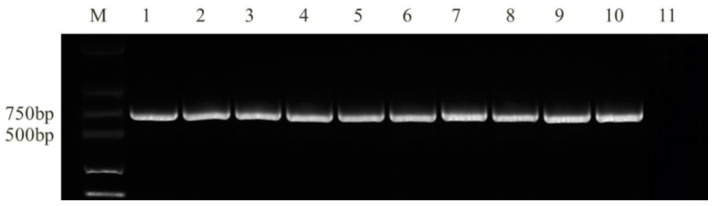
Detection of the gene *FUM1* in *F. proliferatum* isolates using primer Rp32/Rp33 (M, DNA marker; 1–11: D21, D44-2, D56-1, D57-1, D59, D62-1, D65-1, D67, D75, D91, Negative control).

Using the specific primers, the *FUM1* gene was detected in 47 *F. verticillioides* and 19 *F. proliferatum* isolates (Figure 3 and Figure 4). The results showed that these isolates theoretically possessed the capacity to synthesize FBs.

**Figure 5:**
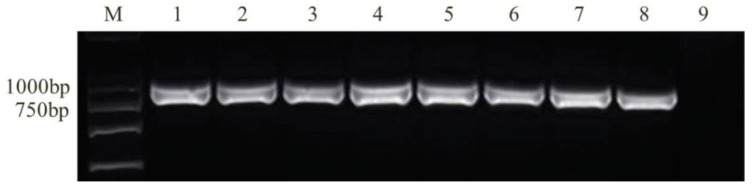
Detection of toxigenic chemotype among FGSC isolates using primer Tri13P1/Tri13P2 (M, DNA marker; 1–9: *Fusarium* spp. CP1, CP5, D38, D66, D66, D73, D76-1, D99, Negative control).

The *Tri13* gene-specific primer Tri13P1/Tri13P2 was used to conduct the PCR amplification of 19 members of the FGSC in Figure 5. A single 859 bp fragment was stably amplified in 16 *F. meridionale* isolates and three *F. asiaticum* isolates, indicating that all 19 members of the FGSC were of the NIV chemotype.

### 2.3. Analysis of FBs

Mycotoxin assays showed that all *F. verticillioides* and *F. proliferatum* isolates could produce toxins FB_1_, FB_2_, and FB_3_. Except for *F. verticillioides* isolates D61-1 and D63, the other isolates exhibited a significantly higher FB_1_ yield than that of FB_2_ and FB_3_ (Table 3 and Table 4). In the *F. verticillioides* isolates, FB production (all toxin production expressed in micrograms per gram of mycelial dry weight in this paper) was 5.76–2015.19 µg/g, with an average of 344.81 µg/g. Among these, the production of toxin FB_1_ ranged from 3.17 to 1566.44 µg/g, with an average of 263.94 µg/g; the production of FB_2_ toxin was between 1.07 and 156.52 µg/g, with an average of 24.70 µg/g; and the production of FB_3_ toxin varied from 1.52 to 356.15 µg/g, with an average of 56.17 µg/g (Table 3).

Among the *F. proliferatum* isolates, the production of FB_1_, FB_2_, and FB_3_ was within the ranges of 97.74–11,100.99 µg/g, 16.01–1554.83 µg/g, and 9.11–381.4 µg/g, with the corresponding averages of 3632.88, 402.31, and 177.78 µg/g, respectively (Table 4).

Table 3 and Table 4 showed the significant differences in FB production among various isolates. For the *F. verticillioides* isolates, 42.6% of the isolates had <100.00 µg/g toxin production, whereas 68.4% of the *F. proliferatum* isolates exhibited >1000.00 µg/g toxin production, indicating that the toxigenicity of *F. proliferatum* in these areas was higher than that of *F. verticillioides* (Table 5).

### 2.4. Determination of the Toxigenicity of FGSC

The DON, ZEN, and NIV assay based on UHPLC-MS/MS were in agreement with the molecular detection of the *Tri13* gene in members of the FGSC. The toxin assay showed that none of the three *F. asiaticum* strains produced the toxins NIV and ZEN, but only the CP5 isolate produced DON with 4.50 µg/g of dry hyphal weight, suggesting that the *F. asiaticum* produces almost no mycotoxins in Chongqing (Table 6).

NIV was detected in 11 out of the 16 *F. meridionale* isolates, which showed a mycotoxin-producing range of 17.40–2597.34 µg/g of dry hyphal weight. ZEN was detected in 11 isolates, and toxin production ranged from 8.35 to 78.57 µg/g. DON was detected in four isolates, i.e., CP1, CP4, D14, and D46, with the toxin production range of 3.38–33.41 µg/g. Isolates D38, D58-2, and D85-1 expressed 3-AcDON toxin, with the corresponding productions of 7.81, 5.83, and 3.10 µg/g, respectively. 15-AcDON was only detected in D38 isolate, with toxin production of 7.90 µg/g. The results show that *F. meridionale* mainly produces NIV, but weakly does ZEN, DON, 3-AcDON or 15-AcDON.

## 3. Discussion

Maize is an important food crop in China, and it is also a significant energy crop and industrial material. Numerous studies have shown that in most countries and regions, *Fusarium* spp. are the main causative pathogens for maize kernel rot. *F. verticillioides* and *F. graminearum* sensu stricto are the predominant species in Huang-Huai-Hai and northeast China [2,33]. However, our study indicated that besides *F. verticillioides*, *F. proliferatum*, and *F. meridionale* were also the predominant pathogens that caused maize ear rot in Chongqing areas, indicating the characteristic composition of pathogenic *Fusarium* species causing maize ear rot in these areas. These discrepancies may be caused by particular environmental conditions. Chongqing is located in the southwest maize growing area of China, and the area is hilly and mountainous, with the highest elevation reaching up to 2800 m. Different ecological zones are present, thereby forming the unique pathogen community.

The warm, rainy, and humid weather conditions are suitable for infection, growth, and reproduction of *Fusarium* spp. in these areas. It is an important factor contributing to the serious maize ear rot. Besides, cropping system is probably also a major factor. In Chongqing areas, crop planting patterns usually incude corn monoculture, wheat and corn rotation, and rice and corn rotation, and so on. Undoubtedly, inoculum production increases with corn monoculture. In addition, *F. verticillioides*, *F. proliferatum*, FGSC, and *F. oxysporum* are also important pathogenic fungi in wheat and rice. Therefore, corn rotation with wheat or rice hardly reduces the prevalence of these fungi, even leading to the accumulation of the above *Fusarium* species in these areas.

FBs, DON, NIV, ZEA, and other mycotoxins are the major causes of toxin contamination by *Fusarium* species. However, both *F. verticillioides* and *F. proliferatum* produce FBs, the former can cause FB contamination mainly in maize, whereas the latter can cause toxin contamination in a variety of crops. Mycotoxin assays showed that all *F. proliferatum* and *F. verticillioides* isolates could produce toxins the FB_1_, FB_2_, and FB_3_, with FB_1_ as the predominant mycotoxin. However, the average toxin production of *F. proliferatum* isolates was 12.22-fold higher than that of *F. verticillioides*, and hence, potential contamination with *F. proliferatum* should always be fully considered. In the present study, PDB liquid medium was used in culturing the *Fusarium* strains, and whether the toxin production of *F. proliferatum* was the highest in vivo will be investigated in our future study. In the field or in storage, mycotoxin contamination from maize ears and kernels is heavily influenced by multiple factors, such as pathogens, environmental conditions (temperature, humidity, pH, and lighting), host resistance, and so on. Therefore, mycotoxin production from these isolates in the laboratory primarily represents their toxigenic potential.

In this study, the results of the Tri13P1/Tri13P2 specific primer assay and toxin detection indicated that 16 *F. meridionale* and 3 *F. asiaticum* isolates were of the NIV chemotype, thereby representing geographical characteristics. Kuppler et al. reported that among 63 FGSC strains from Germany, only two belonged to NIV type, and the remaining were of the DON type [34]. In France, only 14.6% of the members of the FGSC were of the NIV type, and the remaining 85.4% belonged to the 15-ADON type [35]. In Brazil, among the 92 strains of the FGSC isolated from barley, 61 (66.3%), 4 (4.4%) and 27 (29.3%) belonged to 15-ADON, 3-ADON, and NIV chemotype, respectively [31].

In China, studies on the population structure and toxigenicity of the FGSC have mostly focused on wheat and rice, whereas studies on maize are very limited. Shen et al. found that among 530 FGSC strains isolated from the main winter wheat-producing areas of China, 182 *F. graminearum* sensu stricto strains were mainly distributed in North China, and 348 *F. asiaticum* strains were mainly distributed in South China. Among these, a high isolation frequency of the 15-ADON strains was observed in North China, and the NIV and 3-ADON strains were more common in South China [28]. Similar studies also have proven that the NIV and 3-ADON strains are mostly distributed in warmer regions [36,37]. Our findings that FGSC isolates causing maize ear rot in Chongqing areas were of the NIV genotype also support the above conclusions.

Studies have shown that various mycotoxins have different toxicological properties. Compared to the toxin DON, NIV poses a more serious threat to humans and animals health, which requires a more stringent limit of daily intake [38,39]. NIV was detected in 57.9% of the FGSC, and three isolates had a relatively high NIV-producing capacity (>1000 µg/g of dry hyphal weight) were *F. meridionale*. Compared to NIV, these isolates produced a small amount of ZEA and DON toxins. These findings indicate that NIV is likely to be the predominant trichothecene contaminant in Chongqing areas.

In the present study, we found that several *F. meridionale* isolates, such as D58-2, D66, D73, D85-1, and D91-2, and *F. asiaticum* strains D99, CP5, and D57-2 harbored the gene responsible for NIV-production but did not secrete NIV toxin. These phenotypes could be explained by a mutation in the NIV producing gene sequences, or by altered expression of the NIV producing genes. Also, the amount of NIV toxin produced by these isolates is probably beyond the detection limit of our assays. In addition, the lack of NIV in these isolates may also be due to the growth medium used.

Based on our study, various *Fusarium* species show distinct differences in their toxigenicity. Therefore, in Chongqing areas, the potential maize food and feed safety threat caused by *F. proliferatum* and *F. meridionale* is probably more serious than that by *F. verticillioides*, and *F. asiaticum*, respectively. However, maize germplasm and varieties are usually merely screened for resistance to ear rot caused by *F. verticillioides* and *F. graminearum* sensu stricto in China. Therefore, the risk of growing the selected “resistant” varieties remains. Although *F. proliferatum* is not the firstly major causal pathogen of ear and kernel rot, this species should also be included in germplasm screening for resistance and crop breeding for disease resistance, particularly in Chongqing areas. Also, maize ear and kernel rot caused by *F. meridionale* deserves attention. The maize germplasm resistant to *F. meridionale* should be selected for cultivation in these areas. In addition, *F. proliferatum* contamination may be utilized as an important indicator of the quality and safety of grains produced in these particular areas.

## 4. Materials and Methods

### 4.1. Sample Collection and Isolation and Identification of Pathogenic Fungi

**Figure 6:**
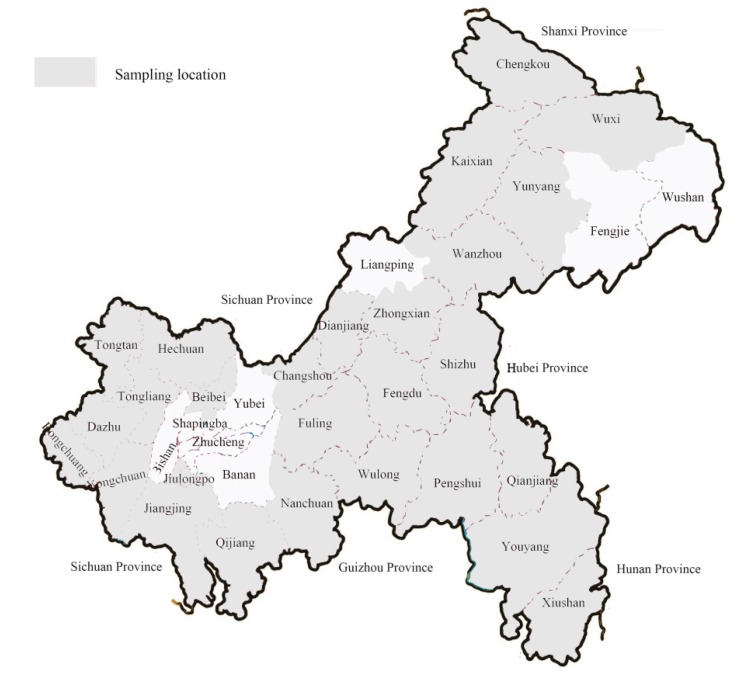
Sampling locations in Chongqing areas.

A total of 103 maize ear or kernel samples (five symptomatic maize ears or 500 g of kernels for each sample) were collected from production fields at harvest in 103 towns of 34 counties in Chongqing and surrounding areas in 2014 and 2015 (Figure 6 and Table S1). About 30 seeds collected from each sample were soaked in 20% sodium hypochlorite solution for 3 min, and rinsed with sterile water thrice. These seeds were dried with sterile filter paper and placed on a potato dextrose agar (PDA) (potato infusion 200 g, dextrose 20 g, agar 20 g, distilled water 1000 mL) plate for culture for 3 days at 25 °C. Hyphae from typical *Fusarium* colonies on PDA were transferred to a fresh poor-nutrient potato dextrose agar (half-PDA) (potato infusion 100 g, dextrose 20 g, agar 20 g, distilled water 1000 mL) plate and the culture was grown for 5 to 7 days. Upon emergence of conidia, a single spore was isolated on PDA by the plate dilution method. Finally, the single spore was transplanted onto a PDA plate to culture single-spore isolates.

### 4.2. Identification of Pathogenic Fungi

The morphological identification of fungal cultures was conducted based on general characteristics and conidial morphology [40]. In order to confirm the morphological identification, genomic DNA was extracted from collected aerial mycelia using the Rapid Fungi Genomic DNA Isolation Kit (SK8230, Sangon Biotech, Shanghai, China) according to the manufacturer’s instruction and were validated by species-specific polymerase chain reaction (PCR) for identification (Table 7).

Each PCR reaction system (20 µL) consisted of a DNA template (2.0 µL), upstream and downstream primers (1.0 µL each), 2× Taq PCR Master Mix (10.0 µL), and ddH_2_O (6.0 µL).

Reactions were performed using a GeneAmp PCR System 9700 thermal cycler (ABI, Norwalk, CT, USA) programmed for 94 °C for 5 min; followed by 35 cycles of 95 °C for 50 s, 58–60 °C for 50 s, and 72 °C for 60 s; and a final extension at 72 °C for 10 min. Electrophoretic analysis of the PCR-amplified products was performed on a 1% agarose gel.

The other *Fusarium* species that could not be determined by species-specific PCR were analyzed using the translation elongation factor (*TEF*)*-1α* gene sequences. TEF-F/R: 5′-ATGGGTAAGGARGACAAGAC-3′/5′-GGARGTACCAGTSATCATGTT-3′ [45]. Each PCR reaction system (50.0 µL) consisted of a DNA template (5.0 µL), upstream and downstream primers (2.5 µL each), 2× Taq PCR Master Mix (25.0 µL), and ddH_2_O (15.0 µL). Reactions were performed using a GeneAmp PCR System 9700 thermal cycler programmed for 94 °C for 5 min; followed by 35 cycles of 95 °C 50 s, 53 °C for 50 s, and 72 °C 60 s; and a final extension at 72 °C for 10 min. The amplified PCR products were bi-directionally sequenced by Sangon Biotech, and the sequences were compared with *Fusarium* sequences in the *Fusarium* Center’s database at Penn State. Using MEGA 5.0 software (ASU, Phoenix, AZ, USA, 2011), a phylogenetic tree was constructed via Test Maximum Likelihood Tree clustering method based on the *TEF-1α* gene sequences, and the bootstrap analysis was performed with 1000 replicates for statistical support of branches.

### 4.3. Molecular Identification of Toxigenic Genes

The detection of the *FUM1* gene was conducted using the specific primers: Fum5F/Fum5R (Fum5F: 5′-GTCGAGTTGTTGACCACTGCG-3′ and Fum5R: 5′-CGTATCGTCAGCATGATGTAGC-3′) for *F. verticillioides* isolates and Rp32/Rp33 (Rp32: 5′-ACAAGTGTCCTTGGGGTCCAGG-3′ and Rp33: 5′-GATGCTCTTGGAAGTGGCCTACG-3′) for all *F. verticillioides* and *F. proliferatum* strains, with an annealing temperature of 60 °C [41,46]. The size of amplified fragments was 890 and 680 bp, respectively. The molecular detection of the toxigenic chemotypes of the FGSC was conducted using specific primers, Tri13P1/Tri13P2 (Tri13P1: 5′-CTCSACCGCATCGAAGASTCTC-3′ and Tri13P2: 5′-GAASGTCGCARGACCTTGTTTC-3′), at an annealing temperature of 58 °C [47]. The sizes of amplified fragments of the NIV, 3-AcDON, and 15-AcDON strains were 859, 644, and 583 bp, respectively. PCR reaction system was earlier described. Electrophoretic analysis of the PCR-amplified products was performed on a 1% agarose gel.

### 4.4. Detection of Mycotoxin Production

Equivalent *Fusarium* spp. were cut from half-PDA and placed in a sterilized conical flask containing 150 mL of potato dextrose broth (PDB). Each fungal isolate was cultured in triplicate, and the sterile liquid medium with no inoculant was used as control. *F. verticillioides* and *F. proliferatum* were grown in a 15 day static culture in PDB with pH 8.0 at 25 °C, and FGSC was grown in a 15 day shaking culture (100*g*) in PDB with pH 3.0 at 25 °C [32]. The inoculated culture medium was filtered with a Whatman GF/A glass fiber filter paper, the filtrate was then stored at −80 °C or sterilized under high pressure, and the hyphae were collected, dried, and weighed.

For all *Fusarium* isolates, 20 mL of the filtrate was collected and used in the toxin assays. Immunoaffinity column purification and HPLC analysis of *F. verticillioides* and *F. proliferatum* were performed to measure FB production [48]. The eluent was dried with nitrogen and dissolved in 1.5 mL of 80% methanol solution. FBs were tested using a C_18_ reverse-phase liquid chromatography/fluorescence detector after *O*-phthaldialdehyde (OPA) derivation and quantified via an external standard method. DON, ZEN, and NIV production of FGSC was determined using UHPLC-MS/MS [49]. Samples were extracted with an 80% acetonitrile water solution, purified via a multifunction decontamination column, isolated via a Waters ACQUITY UPLC BEH C18 chromatographic column, tested by multireaction ion monitoring of quadrupole mass spectrometry, and quantified by an external standard method. Statistical analysis was performed with SPSS 10.0 software (SPSS Inc., Chicago, IL, USA, 2007).

## Figures and Tables

**Table 1:** *Fusarium* species isolated from rotted maize ears and kernels in Chongqing areas.

*Fusarium* spp.	Number of Isolates	Isolation Frequency	Isolate Code
*Fusarium verticillioides*	47	40.2%	D11, D12-1, D12-2, D13, D15, D17, D22, D25, D30-2, D31, D32, D33, D34, D40-2, D42, D45, D50, D52, D54, D58-1, D60, D61-2, D62-2, D63, D64, D68-1, D68-2, D70, D72, D74-1, D77, D78-2, D79-1, D80-2, D81, D83-1, D83-2, D84, D85-2, D87, D88-1, D92-1, D93-2, D95-1, D96-1, D98-2, D100
*F. proliferatum*	19	16.4%	D21, D44-2, D56-1, D57-1, D59, D62-1, D65-1, D67, D68-3, D75, D75-2, D78-1, D79-3, D88-2, D89-2, D90-1, D91, D92-2, D93-1
FGSC	19	16.4%	CP1, CP4, CP5, D14, D38, D44-1, D46, D48, D57-2, D58-2, D59-2, D66, D73, D76-1, D82-1, D85-1, D91-2, D92-3, D99
*F. oxysporum*	14	12.1%	D16, D23, D26, D30-1, D61-1, D61-3, D71-2, D78-3, D79-2, D82-2, D86-2, D93-3, D95-2, D96-2
*F. fujikuroi*	8	6.9%	CP2AH, CP2AZ, D24, D69-2, D7, D90-22, D94, D97-2
*F. equiseti*	4	3.4%	D56-2, D80-1, D89-1, D98-1
*F. culmorum*	2	1.7%	D55, D95-3
*F. incarnatum*	1	0.9%	D71-3
*F. kyushuense*	1	0.9%	D40-3
*F. solani*	1	0.9%	D69-1

**Table 2:** The isolation frequency of *Fusarium* species in different regions.

*Fusarium* spp.	Isolation Frequency				
	Northeast Chongqing	Southeast Chongqing	Central Chongqing	West Chongqing	The Others
*F. verticillioides*	28.57%	42.86%	40.00%	35.48%	91.67%
FGSC	20.00%	14.29%	10.00%	22.58%	0.00%
*F. proliferatum*	20.00%	10.71%	20.00%	19.35%	0.00%
*F. oxysporum*	14.14%	25.00%	20.00%	0.00%	8.33%
*F. fujikuroi*	5.71%	7.14%	10.00%	9.68%	0.00%
*F. equiseti*	5.71%	3.57%	0.00%	3.22%	0.00%
*F. culmorum*	2.86%	0.00%	0.00%	3.22%	0.00%
*F. kyushuense*	0.00%	0.00%	0.00%	3.22%	0.00%
*F. incarnatum*	0.00%	3.57%	0.00%	0.00%	0.00%

Northeast Chongqing: Chengkou, Wuxi, Kaixian, Yunyang, Wanzhou, Zhongxian, Fengdu, Dianjiang; Southeast Chongqing: Shizhu, Wulong, Pengshui, Qianjiang, Jiuyang, Xiushan; Central Chongqing: Beibei, Jiulongpo, Fuling, Changshou; West Chongqing: Tongnan, Hechuan, Tongliang, Dazhu, Rongchang, Yongchuang, Jiangjin, Wansheng, Nanchuan, Qijiang; the others: Bazhong, Neijiang, Yibin, Ziyang, Chengdu, Xichang.

**Table 3:** Mycotoxin production of *F. verticillioides* isolates in Chongqing areas ^1^.

No.	Origin of Isolate	FB_1_ (µg/g)	FB_2_ (µg/g)	FB_3_ (µg/g)	FBs (µg/g)
D100	Jiulongpo	148.56 ± 3.51	15.32 ± 2.53	24.51 ± 2.01	188.39 ± 8.33
D11	Hechuan	21.13 ± 1.32	5.47 ± 1.12	7.71 ± 1.56	34.31 ± 4.01
D12-1	Longyu	18.33 ± 1.41	2.58 ± 0.28	3.22 ± 0.69	24.13 ± 2.11
D12-2	Longyu	584.06 ± 8.53	81.22 ± 0.89	69.98 ± 3.21	735.26 ± 10.35
D13	Suzhou	35.30 ± 2.30	7.88 ± 1.03	10.32 ± 1.11	53.50 ± 2.36
D15	Changping	122.84 ± 4.58	10.25 ± 1.55	26.13 ± 3.28	159.22 ± 5.78
D17	Bazhong	20.07 ± 1.11	3.72 ± 0.56	6.21 ± 0.34	30.01 ± 2.15
D22	Bijie	211.83 ± 2.12	19.45 ± 2.58	39.82 ± 2.01	271.10 ± 5.38
D25	Xifeng	56.23 ± 1.56	6.86 ± 1.13	15.37 ± 0.88	78.46 ± 4.56
D30-2	Pujiang	13.20 ± 0.89	3.41 ± 0.77	6.79 ± 0.67	23.40 ± 1.81
D31	Pujiang	11.49 ± 1.13	2.51 ± 0.56	5.82 ± 0.87	19.82 ± 1.87
D32	Pujiang	3.17 ± 0.33	1.07 ± 0.39	1.52 ± 0.30	5.76 ± 0.68
D33	Pujiang	209.67 ± 4.55	17.93 ± 2.57	47.14 ± 2.56	274.74 ± 6.30
D34	Zizhong	26.86 ± 1.20	4.78 ± 0.46	12.28 ± 1.89	43.92 ± 2.51
D40-2	Handan	968.68 ± 6.38	74.51 ± 5.48	105.01 ± 5.11	1148.19 ± 13.15
D42	Qinhuangdao	19.25 ± 1.09	5.00 ± 0.77	6.34 ± 0.55	30.60 ± 2.33
D45	Luanxian	9.44 ± 0.55	2.17 ± 0.69	2.63 ± 0.51	14.24 ± 1.26
D50	Yibin	90.53 ± 2.37	7.91 ± 0.88	13.93 ± 2.14	112.36 ± 4.23
D52	Yibin	35.91 ± 1.22	1.80 ± 0.20	16.38 ± 1.89	54.10 ± 3.18
D54	Yibin	1076.93 ± 16.78	51.51 ± 4.26	356.15 ± 15.11	1484.59 ± 17.33
D58-1	Qijiang	502.83 ± 6.56	51.41 ± 2.21	200.92 ± 10.23	755.16 ± 7.36
D60	Dianjiang	63.20 ± 2.59	6.14 ± 0.58	10.60 ± 0.95	79.95 ± 3.56
D61-1	Dianjiang	22.22 ± 1.08	8.98 ± 0.39	16.02 ± 1.08	47.21 ± 2.88
D62-2	Nanchuan	270.12 ± 5.02	21.35 ± 2.56	46.02 ± 2.58	337.49 ± 7.77
D63	Nanchuan	10.28 ± 0.56	8.20 ± 0.77	6.83 ± 0.86	25.32 ± 2.03
D64	Changshou	167.70 ± 4.56	8.58 ± 0.95	53.40 ± 3.33	229.68 ± 5.08
D68-1	Rongchang	8.90 ± 0.63	1.62 ± 0.19	4.88 ± 0.88	15.40 ± 1.26
D68-2	Rongchang	283.87 ± 5.17	32.87 ± 2.15	72.44 ± 5.69	389.18 ± 7.02
D70	Rongchang	233.19 ± 5.31	42.68 ± 2.33	145.60 ± 8.12	421.47 ± 5.59
D72	Xiushan	26.26 ± 1.09	3.72 ± 0.69	14.35 ± 2.99	44.33 ± 2.03
D74-1	Shizhu	112.39 ± 4.26	15.73 ± 0.97	57.14 ± 3.11	185.26 ± 5.69
D77	Dazhu	261.92 ± 4.63	25.38 ± 3.33	80.67 ± 4.23	367.98 ± 5.78
D78-2	Youyang	353.08 ± 7.89	22.778 ± 2.68	129.00 ± 5.55	504.86 ± 8.26
D79-1	Youyang	848.51 ± 9.97	42.33 ± 3.15	167.50 ± 6.42	1058.35 ± 10.89
D80-2	Xiushan	1005.51 ± 10.36	86.65 ± 4.13	128.81 ± 5.43	1220.98 ± 10.29
D81	Qianjiang	206.21 ± 5.12	19.69 ± 1.22	30.37 ± 2.33	256.26 ± 5.96
D83-1	Penshui	36.09 ± 2.01	8.24 ± 0.57	10.88 ± 1.46	55.20 ± 2.39
D83-2	Penshui	44.05 ± 2.25	6.39 ± 0.88	12.21 ± 1.39	62.65 ± 2.54
D84	Fumeng	100.87 ± 3.87	8.96 ± 1.09	31.28 ± 3.11	141.11 ± 4.37
D85-2	Pengshui	1566.44 ± 12.66	156.52 ± 5.55	292.22 ± 6.47	2015.19 ± 13.89
D87	Wulong	215.51 ± 7.01	20.83 ± 2.07	47.24 ± 2.11	283.58 ± 8.09
D88-1	Tongnan	37.15 ± 1.17	6.62 ± 0.89	12.51 ± 1.03	56.27 ± 2.15
D92-1	Chengkou	206.02 ± 3.56	6.37 ± 1.22	11.15 ± 1.35	223.54 ± 4.23
D93-2	Chengkou	89.92 ± 3.43	9.18 ± 0.91	25.29 ± 2.10	124.38 ± 3.89
D95-1	Wuxi	749.74 ± 8.01	77.64 ± 3.87	122.98 ± 5.88	950.36 ± 9.52
D96-1	Yunyang	968.97 ± 11.12	95.69 ± 3.60	97.01 ± 4.53	1161.67 ± 13.52
D98-2	Wanzhou	330.59 ± 5.23	41.03 ± 2.11	35.68 ± 3.68	407.30 ± 6.56

^1^ Values are means ± SE.

**Table 4:** Mycotoxin production of *F. proliferatum* isolates in Chongqing ^1^.

No.	Origin of Isolate	FB_1_ (µg/g)	FB_2_ (µg/g)	FB_3_ (µg/g)	FBs (µg/g)
D21	Beibe	3082.95 ± 30.78	231.44 ± 5.22	155.25 ± 5.36	3469.64 ± 33.69
D44-2	Fengdu	5331.14 ± 45.36	463.27 ± 5.68	204.95 ± 5.21	5999.36 ± 47.23
D56-1	Yongchuan	157.23 ± 5.23	31.16 ± 2.11	25.14 ± 1.01	213.52 ± 5.89
D57-1	Qijiang	194.99 ± 6.12	16.01 ± 0.89	18.59 ± 1.53	229.59 ± 6.57
D59	Dianjiang	5947.56 ± 38.12	866.02 ± 9.45	308.96 ± 9.31	7122.54 ± 40.12
D62-1	Nanchuan	5666.14 ± 46.25	229.35 ± 7.36	124.29 ± 5.23	6019.77 ± 47.76
D65-1	Changshou	4578.41 ± 23.39	800.77 ± 9.12	316.49 ± 8.01	5695.67 ± 26.59
D67	Wansheng	1284.52 ± 15.23	101.54 ± 2.89	231.50 ± 5.34	1617.55 ± 17.25
D68-3	Rongchan	366.54 ± 6.55	34.09 ± 3.11	75.35 ± 2.59	475.97 ± 7.67
D75	Zhongxian	9130.53 ± 52.47	867.38 ± 18.12	317.26 ± 7.21	10,315.17 ± 54.28
D75-2	Zhongxian	6357.95 ± 39.58	534.06 ± 6.39	200.89 ± 6.33	7092.89 ± 41.26
D78-1	Youyang	5579.13 ± 37.45	390.65 ± 8.88	258.46 ± 8.77	6228.24 ± 38.97
D79-3	Youyang	632.89 ± 10.24	90.55 ± 3.69	141.27 ± 6.37	864.71 ± 11.25
D88-2	Tongnan	97.74 ± 4.63	16.48 ± 1.10	9.11 ± 0.78	123.33 ± 4.99
D89-2	Kaixian	299.31 ± 6.11	36.92 ± 3.55	19.66 ± 1.25	355.89 ± 7.21
D90-1	Kaixian	936.56 ± 11.56	66.04 ± 2.01	44.61 ± 3.21	1047.21 ± 14.22
D91	Shizhu	2813.66 ± 22.37	881.77 ± 10.56	250.86 ± 6.78	3946.29 ± 26.85
D92-2	Chenkou	11,100.99 ± 56.79	431.62 ± 9.23	293.84 ± 10.57	11,826.45 ± 58.76
D93-1	Chenkou	5466.50 ± 35.76	1554.83 ± 16.37	381.40 ± 9.78	7402.72 ± 38.83

^1^ Values are means ± SE.

**Table 5:** The comparison of mycotoxin production between by *F. verticillioides* and *F. proliferatum* ^1^.

*Fusarium* spp.	FB_1_ (µg/g)	FB_2_ (µg/g)	FB_3_ (µg/g)	FBs (µg/g)
*F. verticillioides*	263.94 ± 4.01 A	24.70 ± 3.75 A	56.1 8 ± 2.95 A	344.81 ± 6.51 A
*F. proliferatum*	3632.88 ± 23.70 B	402.31 ± 6.02 B	177.78 ± 4.51 B	4212.97 ± 25.89 B

^1^ Values are means ± SE. The values with the different capital letter in the column express extremely significant difference (*p* < 0.01), according to Duncan’s multiple range test.

**Table 6:** Mycotoxin chemotype and production of FGSC isolates in Chongqing ^1^.

No.	Species ^2^	Origin	Genotype	NIV (µg/g)	DON (µg/g)	15-ADON (µg/g)	3-ADON (µg/g)	ZEN (µg/g)
CP1	*F. m.*	Fuling	NIV	699.55 ± 11.23	19.43 ± 1.56	0.00	0.00	0.00
CP4	*F. m*.	Jiangjin	NIV	1254.86 ± 18.68	3.77 ± 0.38	0.00	0.00	0.00
D14	*F. m.*	Wanzhou	NIV	2597.34 ± 25.48	33.41 ± 2.69	0.00	0.00	8.35 ± 0.67
D38	*F. m.*	Jiangjin	NIV	143.52 ± 5.36	0.00	7.90 ± 0.89	7.81 ± 0.57	12.72 ± 1.03
D44-1	*F. m.*	Fengdu	NIV	1004.84 ± 13.89	0.00	0.00	0.00	14.57 ± 1.25
D46	*F. m.*	Chengkou	NIV	450.11 ± 8.37	3.38 ± 0.56	0.00	0.00	0.00
D48	*F. m.*	Chengkou	NIV	89.25 ± 3.21	0.00	0.00	0.00	78.57 ± 3.89
D58-2	*F. m.*	Qijiang	NIV	0.00	0.00	0.00	5.83 ± 0.67	56.40 ± 4.25
D59-2	*F. m.*	Dianjiang	NIV	123.29 ± 5.87	0.00	0.00	0.00	0.00
D66	*F. m.*	Wansheng	NIV	0.00	0.00	0.00	0.00	0.00
D73	*F. m.*	Shizhu	NIV	0.00	0.00	0.00	0.00	49.06 ± 3.58
D76-1	*F. m.*	Dazhu	NIV	90.89 ± 4.21	0.00	0.00	0.00	71.68 ± 3.79
D82-1	*F. m.*	Qianjiang	NIV	17.40 ± 1.56	0.00	0.00	0.00	51.65 ± 2.87
D85-1	*F. m.*	Wulong	NIV	0.00	0.00	0.00	3.10 ± 0.22	38.95 ± 3.19
D91-2	*F. m.*	Shizhu	NIV	0.00	0.00	0.00	0.00	31.57 ± 2.45
D92-3	*F. m.*	Chengkou	NIV	61.87 ± 3.05	0.00	0.00	0.00	42.38 ± 2.71
D99	*F. a.*	Wanzhou	NIV	0.00	0.00	0.00	0.00	0.00
CP5	*F. a.*	Tongliang	NIV	0.00	4.50 ± 0.55	0.00	0.00	0.00
D57-2	*F. a.*	Qijiang	NIV	0.00	0.00	0.00	0.00	0.00

^1^ Values are means ± SE; ^2^ *F. m.* = *F. meridionale*; *F. a.* = *F. asiaticum*.

**Table 7:** Specific primer pairs for *Fusarium* spp.

Fungi	Primer	Sequences (5′–3′)	Product Size (bp)	*T_m_* (°C)	Reference
*Fusarium* spp.	ItsF	AACTCCCAAACCCCTGTGAACATA	431	58	[41]
	ItsR	TTTAACGGCGTGGCCGC			
FGSC	Fg16NF	ACAGATGACAAGATTCAGGCACA	280	57	[42]
	Fg16NR	TTCTTTGACATCTGTTCAACCCA			
*F. oxysporum*	FoF1	ACATACCACTTGTTGCCTCG	340	58	[43]
	FoR1	CGCCAATCAATTTGAGGAACG			
*F. verticillioides*	VER1	CTTCCTGCGATGTTTCTCC	578	56	[44]
	VER2	AATTGGCCATTGGTATTATATATCTA			
*F. proliferatum*	PRO1	CTTTCCGCCAAGTTTCTTC	585	56	[44]
	PRO2	TGTCAGTAACTCGACGTTGTTG			

## Supplementary Materials

The following are available online at http://www.mdpi.com/2072-6651/10/2/90/s1, Table S1: Sampling information.

## Abbreviations

The following abbreviations are used in this manuscript:

3-ADON	3-acetyl-deoxynivalenol
15-ADON	15-deoxynivalenol
DON	deoxynivalenol
FB	fumonisin B
HPLC	high performance liquid chromatography
NIV	nivalenol
OPA	*O*-phthaldialdehyde
PCR	polymerase chain reactions
PDA	potato dextrose agar
PDB	potato dextrose broth
SNA	Spezieller Nährstoffarmer agar
*TEF*	translation elongation factor
UHPLC-MS/MS	ultra-high performance liquid chromatography-mass spectrometry
ZEN	zearalenone
