# Supplementary material for: The Major Fusarium Species Causing Maize Ear and Kernel Rot and Their Toxigenicity in Chongqing, China
Source: Toxins (Basel). 2018 Feb 22;10(2):90. doi: 10.3390/toxins10020090 (PMC5848190; doi:10.3390/toxins10020090)
Supplement: Supplementary file 1 [file toxins-10-00090-s001.pdf]

Supplementary Materials: The Major *Fusarium* Species Causing Maize Ear and Kernel Rot and Their Toxigenicity in Chongqing, China

Danli Zhou, Xiaoming Wang, Guokang Chen, Suli Sun, Yang Yang, Zhendong Zhu and Canxing Duan

Table S1. Sampling information.

Sample Location	Number of Samples	Elevation (m)	Average Precipitation (mm)	Sample Year
Beibei	9	175-1312m	1201	2014
Hechuan	3	185-1285	1213	2014
Xiushan	6	246-1631	1323	2014
Fulin	1	138-1977	1090	2014
Jiangjin	6	179-1709	1035	2014
Chengkou	3	482-2686	1246	2014
Tongliang	1	185-902	1068	2014
Wanzhou	1	106-1762	1211	2014
Yongchuan	4	180-420	1045	2014
Fengdu	4	175-2000	1080	2014
Bazhong	2	267-2513	1124	2014
Chengdu	4	380-3000	1100	2014
Neijiang	4	300-500	1035	2014
Xichang	1	1500-2500	1088	2014
Ziyang	2	300-550	955	2014
Yibing	6	270-1418	1168	2014
Yunyang	1	139-1809	1118	2015
Xiushan	3	246-1631	1350	2015
Youyang	2	263-1895	1356	2015
Chengkou	2	482-2686	1263	2015
Dianjiang	3	320-1183	1179	2015
Jiulongpo	1	180-450	1056	2015
Nanchuan	2	340-2251	1156	2015
Qijiang	2	188-1973	1076	2015
Rongchang	3	300-711	1119	2015
Wansheng	2	265-1973	1303	2015
Wulong	2	160-2033	1079	2015
Shizhu	3	119-1934	1112	2015
Zhongxian	1	117-1680	1185	2015
Tongnan	2	300-450	975	2015
Wanzhou	3	106-1762	1194	2015
Wuxi	2	139-2796	1140	2015
Yongchuan	2	180-420	1089	2015
Changshou	2	300-1034	1145	2015
Dazu	2	270-934	1007	2015
Kaixian	2	134-2626	1228	2015
Pengshui	2	612-1084	1227	2015
Qianjiang	2	320-1939	1203	2015
